# A review of the cavernicolous genus *Guiaphaenops* Deuve, with the description of a new species (Coleoptera, Carabidae, Trechinae)

**DOI:** 10.3897/zookeys.669.12334

**Published:** 2017-04-20

**Authors:** Bin Feng, Guofu Wei, Mingyi Tian

**Affiliations:** 1 Forestry Department of Guangxi Zhuang Autonomous Region, No. 21, Yunjing Road, Nanning, 530028, China; 2 Administrative Bureau of Huangjiang World Natural Heritage, Guangxi, 547100, China; 3 Department of Entomology, College of Agriculture, South China Agricultural University, No. 483, Wushan Road, Guangzhou, 510642, China

**Keywords:** China, Guangxi, ground beetle, new species, semi-aphaenopsian, subterranean

## Abstract

The subterranean ground beetle genus *Guiaphaenops* Deuve, 2002 is taxonomically reviewed. This poorly known genus is different from *Guizhaphaenops* Vigna Taglianti, 1997 in having convex propleura which is visible from above and elytral chaetotaxy especially the humeral group of the marginal umbilicate series, in which the 1^st^ pore is transversely and backwardly shifted. The second species, *G. deuvei* Tian, Feng & Wei, **sp. n.**, is described from a limestone cave at Yangli Cun (Village), Lingyun Xian (County), Baise Shi (Prefecture), northwestern Guangxi Zhuang Autonomous Region, China. A key to the species and a distribution map of *Guiaphaenops* are also provided.

## Introduction

Karstic landscapes are diverse in Guangxi Zhuang Autonomous Region, covering more than 42% of the total terrestrial area and having more than 60,000 caves (Zhang et al. 2011). Accordingly, the cave biodiversity is very rich in this region (Tian et al. 2011). For example, more than 30 cave-adapted trechine species belonging to 15 genera have been recorded, the majority of which are members of highly modified aphaenopsian genera, such as *Giraffaphaenops* Deuve, 2002, *Dongodytes* Deuve, 1993, *Pilosaphaenops* Deuve & Tian, 2008 (Tian 2010), *Sinaphaenops* Uéno & Wang, 1991 and *Uenotrechus* Deuve & Tian, 1999 (Tian et al. 2016).

Guiaphaenops Deuve, 2002 was established as a subgenus of Guizhaphaenops Vigna Taglianti, 1997 to arrange *Guiaphaenops lingyunensis* Deuve, 2002, a semi-aphaenopsian species (Deuve 2002). Uéno (2006) treated *Guiaphaenops* as an independent genus considering the peculiar characters of its prothorax and elytral chaetotaxy, and he added another locality cave for *G. lingyunensis*. Until today, *Guiaphaenops* is still a mono-specific genus, known only from two limestone caves in Lingyun Xian, northwestern Guangxi.

In recent years, more material of *Guiaphaenops* has been collected by SCAU team during the cave biological surveys carried out in Lingyun. The findings make it possible to contribute to the knowledge of this interesting genus by providing the new record for *G. lingyunensis* and the description of a new species.

## Material and methods

The blind beetles for this study were collected visually using an aspirator and preserved in 50% ethanol before study. All specimens are deposited in the insect collection of South China Agricultural University, Guangzhou, China (**SCAU**).

Techniques, terminology and abbreviations used in the text follow Tian et al. (2016).

## Taxonomic treatments

### Genus Guiaphaenops

Taxon classificationAnimaliaColeopteraCarabidae

Deuve, 2002

Subgenus Guiaphaenops (of Guizhaphaenops Vigna Taglianti, 1997), Deuve, 2002: 516 (type species: Guizhaphaenops lingyunensis Deuve, 2002).
Genus Guiaphaenops , Uéno, 2006: 22

#### Main generic characteristics.

Median sized and semi-aphaenopsian beetles, eyeless and depigmented; appendages rather long, antennae extending at (female) or over (male) elytral apices; dorsal surface glabrous though a few short hairs present on genae; fore part including mandibles nearly as long as elytra. Head rather elongated, much longer than wide, sub-tubiform; genea slightly and gradually narrowed posteriorly, frontal furrows uncompleted, effaced posteriorly, presence of two pairs of frontal setiferous pores; mandibles thin and elongated, feebly curved apically, right mandibular teeth bidentate; mentum and submentum fused, mental tooth simple, base of mentum distinctly concave, submentum 8- to 10-setose. Prothorax evidently wider than head, propleura distinctly convex and evidently visible from above; pronotum sub-quadrate, slightly wider than head, evidently longer than wide, presence of two pairs of latero-marginal setae, side margins slightly or strongly sinuate before hind angles which are more or less broadly lobed. Elytra sub-ovate, much wider than prothorax, shoulders rounded, prehumeral borders arcuate or nearly oblique, lateral margins ciliate in basal half; striae lacking though somewhat traceable; presence of two dorsal pores and the preapical pore on each elytron. Chaetotaxy: the 1^st^ pore in the humeral group of the marginal umbilicate series transversely and backwardly shifted, at level behind the 2^nd^ pore; the 5^th^ and 6^th^ pores in the middle group close to each other. Protibia smooth, without longitudinal sulcus; only the 1^st^ protarsomere modified in male. Abdominal ventrite VII bisetose in male, while quadrisetose in female. Male genitalia weakly sclerotized, very small, slightly curved ventrally in lateral view, with a quite large sagittal aileron; apical lobe broad in dorsal view; parameres moderately developed, each with 4 rather short apical setae.

#### Discussion.

Though *Guiaphaenops* is more or less similar to the genus *Guizhaphaenops*, the peculiar characteristics such as propleura of prothorax convex and visible from above and the 1^st^ pore of elytral marginal umbilicate series transversely and backwardly suggest that it has to be isolated from the latter genus (Uéno, 2006). *Guiaphaenops* is probably closer to *Zhijinaphaenops* Uéno & Ran, 2002 than to *Guizhaphaenops* in a strict sense because the above mentioned morphological features of *Guiaphaenops* are also shared by *Zhijinaphaenops*. However, *Guiaphaenops* is easily distinguished from the latter genus by its glabrous and smooth body (wholly pubescent in *Zhijinaphaenops*), roundly lobed hind angles (well-marked in *Zhijinaphaenops*), presence of anterior frontal pores on head and hind latero-marginal setae on pronotum (both absent in *Zhijinaphaenops*), and sub-ovate elytra (elongated ovate in *Zhijinaphaenops*).

#### Range.

**Figure 1.**
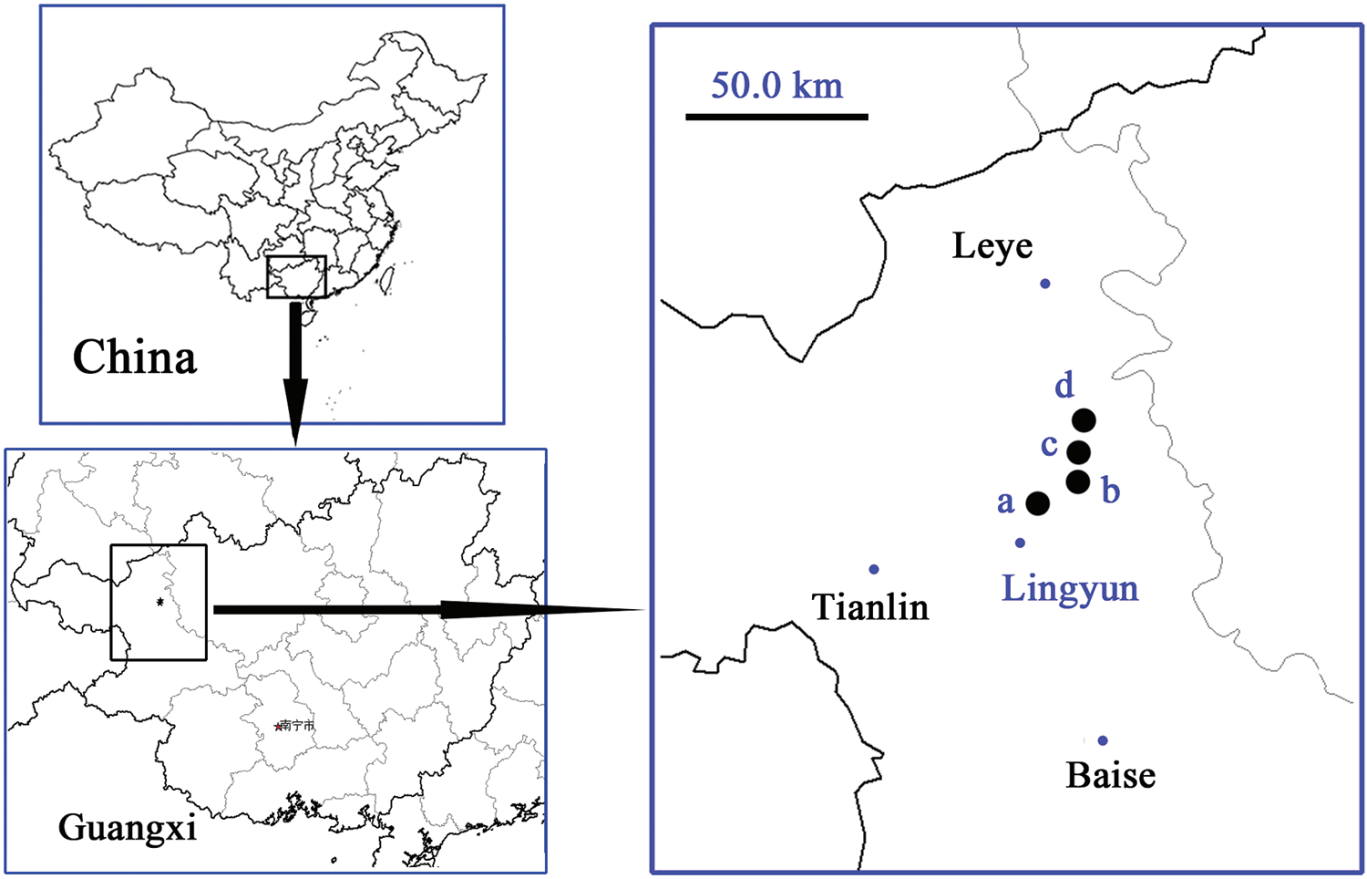
Distribution of *Guiaphaenops* species. **a, b, d** *G. lingyunensis* Deuve **c** *G. deuvei* Tian, Feng & Wei, sp. n.

China (Guangxi). Known only by two species from four limestone caves in Lingyun Xian (Fig. 1).

#### Key to species of Guiaphaenops

**Table:** 

1	Latero-margins of pronotum strongly sinuate before hind angles (Fig. 3b), elytral prehumeral borders nearly oblique (Fig. 4b)	***G. deuvei* Tian, Feng & Wei, sp. n.**
–	Latero-margins of pronotum slightly sinuate before hind angles (Fig. 3a), elytral prehumeral borders distinctly arcuate (Fig. 4a)	***G. lingyunensis* Deuve, 2002**

### Guiaphaenops deuvei

Taxon classificationAnimaliaColeopteraCarabidae

Tian, Feng & Wei sp. n.

http://zoobank.org/623D0DFD-CA91-4AE0-9418-99D1F7B59C83

**Figure 2.**
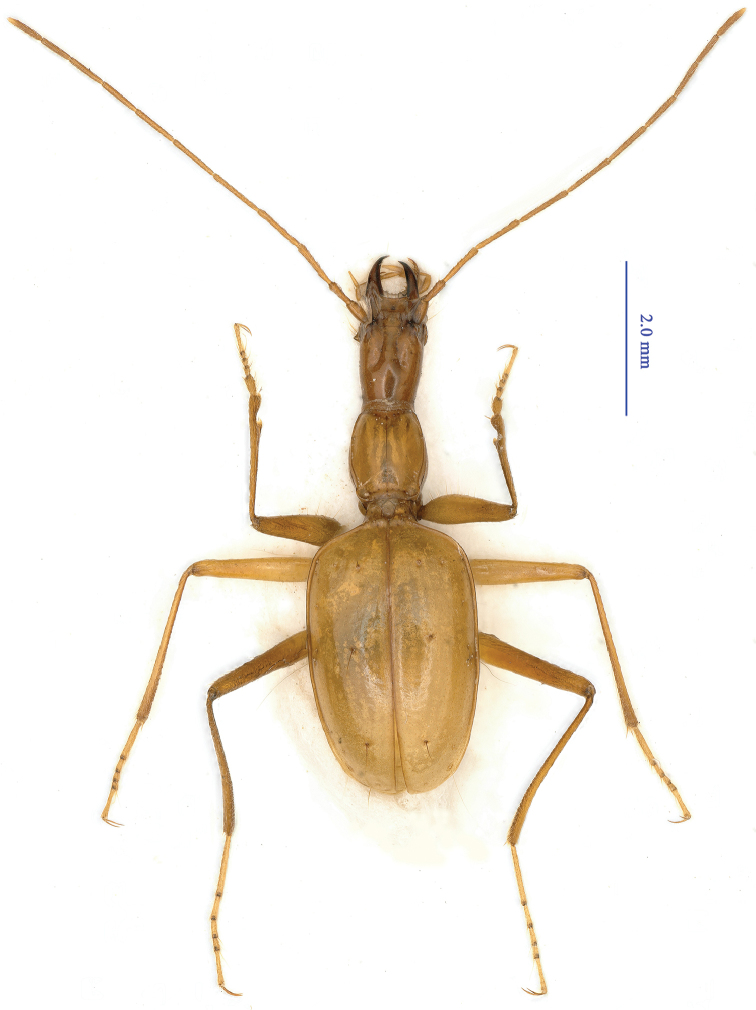
Habitus of *Guiaphaenops deuvei* Tian, Feng & Wei, sp. n., holotype, male.

**Figure 3.**
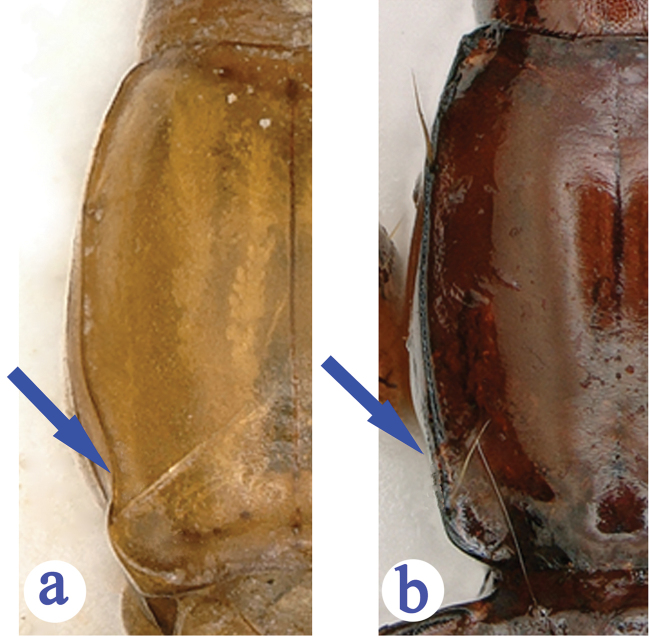
Pronota of *Guiaphaenops* species. **a** *G. deuvei* Tian, Feng & Wei, sp. n. **b** *G. lingyunensis* Deuve.

**Figure 4.**
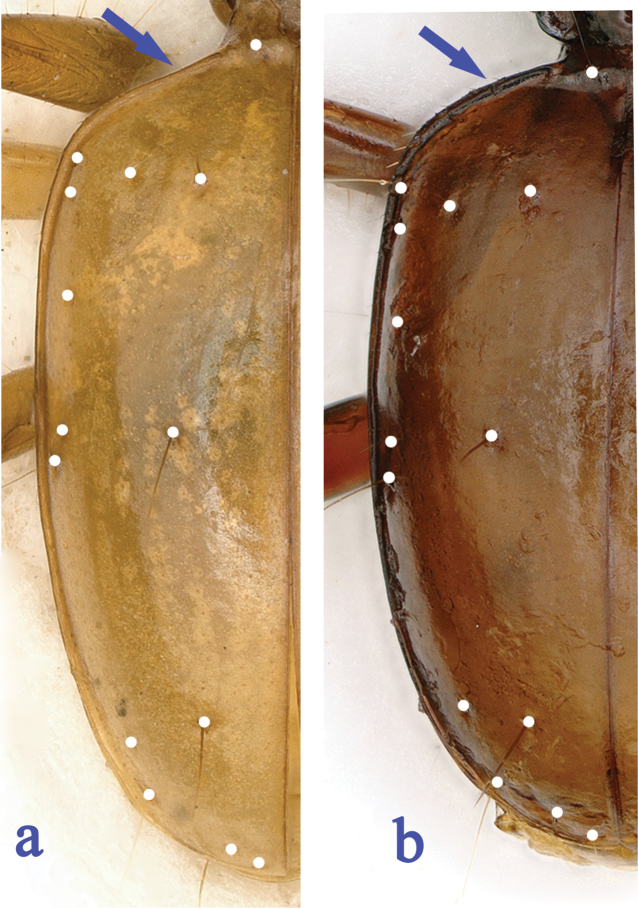
Left elytra of *Guiaphaenops* species, chaetotaxy shown by white points. **a** *G. deuvei* Tian, Feng & Wei, sp. n. **b** *G. lingyunensis* Deuve.

**Figure 5.**
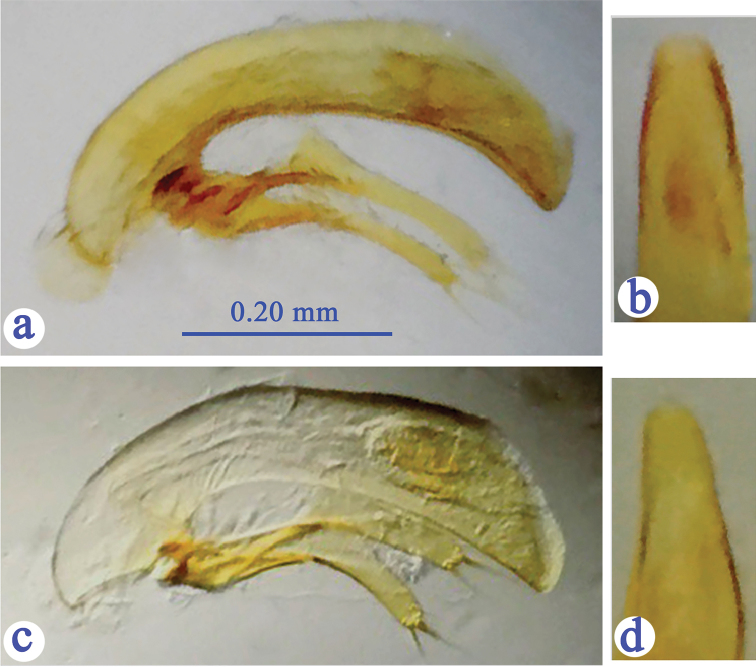
Male genitalia of *Guiaphaenops* species, median lobe and parameres, lateral view (**a, c**) and apical lobe, dorsal view (**b, d**) (**a, b** *G. deuvei* Tian, Feng & Wei, sp. n. **c, d** *G. lingyunensis* Deuve).

**Figure 6.**
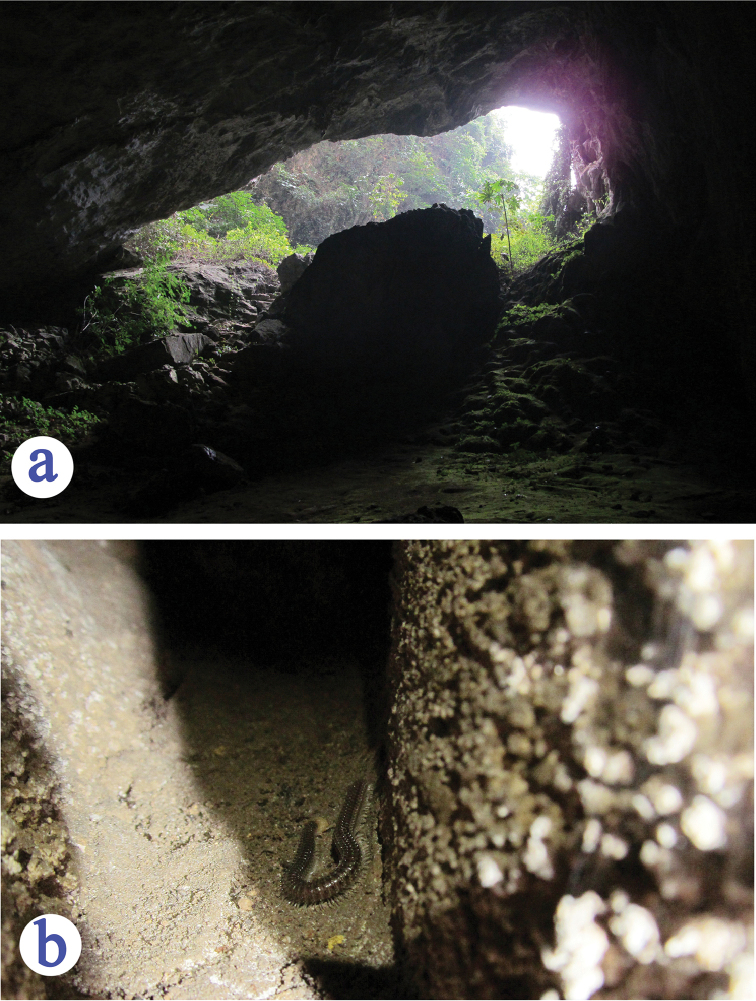
The type locality cave of *Guiaphaenops deuvei* Tian, Feng & Wei, sp. n. **a** cave entrance **b** a millipede in cave.

Figs 1c , 2 , 3a , 4a , 5a, b , 6

#### Holotype.

male, an anonymous cave near Yangli Cun, Jiayou Zhen, Lingyun Xian, Baise Shi, Guangxi, 24°28'39"N, 106°37'52"E, 643 m, VII-25-2012, Mingyi Tian, Weixin Liu, Feifei Sun & Haomin Yin leg., in South China Agricultural University, Guangzhou, China (SCAU).

#### Diagnosis.

A larger species, latero-margins of pronotum strongly sinuate before hind angles which are distinctly lobed and reflexed (Fig. 3a); the 1^st^ pore of the humeral set of umbilicate pores at level before anterior dorsal pore, while the 7^th^ pore behind the level of the preapical pore (Fig. 4a); the median lobe of aedeagus a little slenderer, with apical lobe narrowly constricted towards apex in dorsal view (Fig. 5a, b).

#### Description.

Length: 7.0 mm, width: 2.0 mm. Fore body (including mandibles) shorter than elytra. Habitus as in Fig. 2.

Yellowish brown, with pale appendages. Moderately shiny. Body smooth and glabrous, except genae, underside of head and prosternum each with several sparse setae, ventrites IV-VI with a few short setae between paramedian setae. Microsculptural engraved meshes finely transverse striated.

Head much longer than wide (HLm/HW = 2.45, HLl/HW = 1.75); genae fairly developed, slightly dilated laterally, slightly constricted posteriorly until the well-marked neck constriction; widest at about middle of head from labrum to base; frons and vertex convex, frontal furrows deep and well-marked, divergent at base and apex, ended a little behind the widest part; clypeus transverse, quadrisetose; labrum transverse, frontal margin more or less serrate, 6-setose; anterior and posterior frontal setiferous pores located at about middle of head from labrum to neck constriction and 1/3 from base to labrum respectively; ligula well developed, bisetose at apex; submentum 8-setose. Antennae long, the 1^st^ antennomere stouter than other, slightly shorter than the 2^nd^ which is slightly shorter than the 11^th^; the 5^th^ and 6^th^ longest, each about twice as long as the 1^st^, then gradually shortened towards the 10^th^.

Prothorax (Fig. 3a) shorter than head (PrL/HLm = 0.55, PrL/HLl = 0.77), widest at about 1/3 from base, longer than wide (PrL/PrW = 1.08), wider than head (PrW/HW = 1.25), slightly wider than pronotum (PrW/PnW = 1.11), much narrower than elytra (PrW/EW = 0.46). Pronotum (Fig. 3a) longer than wide (PnL/PnW = 1.20), and wider than head (PnW/HW = 1.13), base wider than front (PbW/PfW = 1.29); lateral sides and finely bordered throughout, base and front unbordered; lateral margin slightly expanded medially, widest at a little behind middle, strongly sinuate before hind angles which are broadly lobed, fore angle obtuse; latero-marginal setae at about 1/4 of pronotum from front and a little before hind angles respectively. Scutellum small.

Elytra (Fig. 4a) longer than head plus prothorax (EL/(HL+PrL) = 1.21), longer than wide (EL/EW = 1.71); widest at about middle, prehumeral borders evidently ciliate, and nearly oblique; disc moderately convex; striae present though superficial. Chaetotaxy: anterior and posterior dorsal pores at about 1/5 of elytra from base and middle of elytra, preapical pore at 1/6 of elytra from apex, much closer to suture than to apical margin; the 1^st^ pore of the humeral set of umbilicate pores located before anterior dorsal pore, while the 7^th^ pore behind the preapical.

Legs thin and rather long, the 1^st^ tarsomere as long as the 2^nd^–4^th^ tarsomeres together in fore, but longer in middle and hind legs.

Male genitalia (Fig. 5a, b): Weakly sclerotized, small but stouter than in *G. lingyunensis*, apical lobe narrower at apical part.

#### Etymology.

Dedicated to Dr. Thierry Deuve of the National Museum of Natural History, Paris, a well-known carabidologist who has described many new ground beetles of China.

#### Distribution.

China (Guangxi) (Fig. 1c). Known only from the limestone cave near Yangli, Jiayou, Lingyun Xian. This cave opens at bottom of a hill near Yangli Cun on the right side of the road from Lingyun to Leye. It is short cave and the large part inside was dry. The single type specimen was collected in a small wet area. Other cave animals observed in this cave were millipedes, crickets and bats.

### Guiaphaenops lingyunensis

Taxon classificationAnimaliaColeopteraCarabidae

Deuve, 2002

Figs 1 , 3b , 4b , 5c, d

Guiaphaenops lingyunensis Deuve, 2002: 518 (type locality: Cave Shen Dong); Uéno, 2006: 24

#### Diagnosis.

A smaller species, latero-margins of pronotum slightly sinuate before hind angles (Fig. 3b); elytra with prehumeral borders broadly arcuate, the 1^st^ pore of the humeral set of umbilicate pores at level behind anterior dorsal pore, while the 7^th^ pore before level of the preapical pore (Fig. 4b); the median lobe of aedeagus slenderer and more elongated than in *G. deuvei* sp. n., with apical lobe thinner in dorsal view (Fig. 5c, d).

#### Material studied.

1 male, X-14-2015, cave Mi Dong, Mawang Cun, Sicheng Zhen, Lingyun Xian, Baise, Guangxi, 24°24'20"N, 106°35'52"E, 410 m, XII-9-2015, Mingyi Tian & Jujian Chen leg., in SCAU; 1 female, ibid, VI-9-2015, Mingyi Tian, Weixin Liu, Xinhui Wang & Minruo Tang leg., in SCAU.

#### Distribution.

China (Guangxi). Known from three caves (Shen Dong, Mi Dong and a cave near Dazai Tun) in Lingyun Xian (Deuve 2002; Uéno 2006) (Fig. 1a, b, d).

Mi Dong is located at about one kilometre from Mawang Cun, in a valley below the main road from Lingyun to Leye. It opens above a path from the village to Sha Dong, a deeper and larger cave nearby. It is short, after 20 m from the entrance there is a large and complete dark room of 30–50 m in diameter. Majority part of this room was muddy or wet. The two beetle specimens were found quickly running on the wet ground. Other cave animals observed in Mi Dong were two species of millipedes and a bat.

## Supplementary Material

XML Treatment for Guiaphaenops

XML Treatment for Guiaphaenops deuvei

XML Treatment for Guiaphaenops lingyunensis
